# Reflecting on 2100 Days at the Helm of Journal of Extracellular Vesicles: A Thank You Editorial

**DOI:** 10.1002/jev2.70096

**Published:** 2025-06-02

**Authors:** Jan Lötvall

**Affiliations:** ^1^ Krefting Research Centre University of Gothenburg Sweden

As I have stepped down as Editor‐in‐Chief of the *Journal of Extracellular Vesicles (JEV)* on April 30th, 2025, exactly 2100 days after assuming the role on August 1st, 2019, I find myself reflecting on the extraordinary journey of growth and increased understanding of EV biology, that I have had the honour to share with many others within the extracellular vesicle (EV) community. Leading this journal has been one of the most rewarding experiences of my scientific career, and I am deeply grateful to the authors, the editorial team, reviewers and our readers who have contributed to its growth and success.

When I took on this role, the field of EV research, and *JEV*, was in a rapidly expanding phase, and the journal had just months prior received its first impact factor. However, during the past 5 years, the growth of the field and the journal is further accelerating, and we have experienced a deepened understanding of EV biology, in parallel with technological advancements in both the isolation and characterisation of EVs. *JEV* has, from its first inception in 2012, been at the forefront of these developments, publishing groundbreaking studies that have shaped the discourse and pushed the boundaries of our discipline, and is still on a rapidly growing trajectory.

Some highlights from the last 2100 days include:

- The expansion of *JEV*’s scope to embrace not only basic EV research but also clinical translational research, including results of therapeutic clinical trials, shows that the field is rapidly moving towards clinical utilisation of EVs. This also established the journal as a home for both fundamental discoveries and applied innovations in the field.
- The scientific evaluation of close to 4000 submissions to the journal, supported by the hard work of the journal's editorial structure of Deputy Editors and Associate Editors.
- The publication of more than 670 articles in the journal, a vast majority being original research.
- Full‐time employment of Sarah William by ISEV, our Executive Scientific Editor.

Employing Sarah has, without question, been the most important improvement in the journal's management in the last 5 years, as she professionally manages and oversees the journal, far beyond the scope of what part‐time Editors are capable of. Thank you for joining JEV Sarah!

The journal would not be what it is without the dedication of our editorial team, the rigor of our peer reviewers, as well as the trust of the scientific community that submit their work to the journal. Before the employment of Sarah Williams, I have for some periods had the administrative editorial support of several well‐established researchers in the field, including Cecilia Lässer and Deborah Goberdhan. In addition, the crucial role of the Deputy Editors as well as Associate Editors in further scrutinising submissions, is essential for any journal. During my tenure, I have had the support of multiple Deputy Editors, including Simon Powis, Esther Nolte‐‘t Hoen, Hubert Yin, Mary Bebawy, Michael Freeman, Roosmarijn Vandenbroucke, Yong Song Gho, An Hendrix, and recently also Cherie Blenkrion, Simon Swift, Owen Davis and Dolores Di Vizio, and a broad group of Associate Editors. As EiC, I implemented a multi‐step approach to assessing manuscripts, requiring at least two pairs of eyes evaluating a manuscript before the journal declines consideration of a submission, and multiple other assessments by Editors and Reviewers before any work is found acceptable for publication. To retain a reasonable and fair assessment of submissions, I have worked under the principle that one person should be at the helm of the journal, implementing one clear editorial vision for which work is suitable for the journal. For me, the overarching goal for JEV during my tenure has been that any published work should be of ‛general interest to the EV community’, and not only to a niche group of scientists or those only interested in a single disease. And, of course, all published work should be of good quality, where conclusions are supported by the data.

I took over the editorship from the three previous EiCs, Clotilde Thery, Yong Song Gho and Peter Quesenberry, having served in the role for more than 7 years from 2012 to 2019. I am handing over to three new EiCs, Hang Hubert Yin, Pascale Zimmermann and Jennifer Jones. Despite any shortcomings of multiple ‛bosses’, it might be suitable for the journal to have more than one Editor‐in‐Chief at this crucial time, when the journal is growing exponentially. Indeed, the number of submissions to the journal is on a trajectory to reach significantly higher than 1000 in 2025, which reflects growth in excess of 20% compared to 2024. With this increased interest in the journal, I suspect that, in time, the journal will need additional staff, beyond now only Sarah Williams, to sustain speedy and professional assessments of publications.

When writing this, I also wish to acknowledge and mention several individuals in the ISEV leadership who have been supportive and helpful to the journal and me in my role. This includes Past President Andrew Hill, who was President of ISEV when I took on the task as EiC of JEV, as well as his successors, Clotilde Théry and Edit Buzás. Also, I felt strong support from three Executive Chairs at the ISEV board level, with the ISEV journal as their responsibilities, including Susmita Sahoo, Ana Claudia Torrecilhas and Metka Lenassi.

Journal publishing and management is not without its challenges, and as a growing, society‐owned journal, it is important that both the society and the team of editors always strive to be at the forefront of ethical publishing. Through our current publisher, Wiley, JEV is formally a member of the Committee on Publication Ethics (COPE). COPE's framework is a gold standard for ethical publishing, providing critical guidance on authorship disputes, plagiarism, data integrity, conflicts of interest and editorial independence from the owner's influence. I, as EiC of JEV, have always upheld these principles in practice. As the journal and society grow, I strongly suggest that the time has come for ISEV and its journals to formalise their policies and procedures in line with both COPE, and other relevant guidelines. I believe it would further solidify *JEV*’s credibility and independence from outside influence and will reassure authors, reviewers and readers of its dedication to the highest ethical publication standards.

As I pass the baton to my successors, I do so with confidence that *JEV* will continue to thrive. The future of EV research is bright, with uncharted territories awaiting exploration, including the dissection of single vesicle characteristics, improved understanding of biological functions, novel therapeutic applications and the refinement of diagnostic tools. I look forward to witnessing the next chapter of *JEV*’s journey, but now as a reader and supporter, rather than as its steward.

To the EV community and all JEV authors during my period as EiC: Thank you for the privilege of serving you. Science is a cooperative endeavour, and it has been an honour to play a small part in your work. It is important to remember that a journal is nothing better than the work it receives, which is why the authors are the most important contributors. Although my time as Editor‐in‐Chief has concluded, my passion for EV research remains fully committed, and I am sure our paths will cross in the future.

With gratitude and positivity,

**Jan Lötvall**

## Data Availability

Data sharing not applicable to this article as no datasets were generated or analysed during the current study.

